# Concerns, perceived impact, practices, preventive measures, and stress among healthcare workers during COVID-19 pandemic in Malaysia

**DOI:** 10.3389/fpubh.2023.1028443

**Published:** 2023-03-02

**Authors:** Muhammad Alfatih Pahrol, Rohaida Ismail, Nadia Mohamad, Yin Cheng Lim, Rosnawati Muhamad Robat, Sakshaleni Rajendiran, Syahidiah Syed Abu Thahir, Ameerah Su'ad Abdul Shakor, Nurfatehar Ramly, Rafiza Shaharudin

**Affiliations:** ^1^Environmental Health Research Centre, Institute for Medical Research, National Institutes of Health, Ministry of Health of Malaysia, Shah Alam, Malaysia; ^2^Department of Social and Preventive Medicine, Faculty of Medicine, University of Malaya, Kuala Lumpur, Malaysia; ^3^Surveillance and Preparedness Unit, Public Health Division, Selangor State Health Department, Ministry of Health Malaysia, Shah Alam, Malaysia; ^4^Department of Community Health, Faculty of Medicine, Universiti Kebangsaan Malaysia, Kuala Lumpur, Malaysia

**Keywords:** healthcare workers, concerns, perceived impact, practices, preventive measures, stress, COVID-19 pandemic

## Abstract

**Introduction:**

Healthcare workers (HCWs) have been continually exposed to patients with COVID-19 and are at higher risk of contracting the disease. Their psychological health is important for overall wellbeing and productivity, which could lead to a reduction in human errors during the pandemic crisis. This study aimed to measure the level of concerns, work practices, adequacy of preventive measures among HCWs, and the impacts on their life and work, including mental health status during the second wave of the COVID-19 pandemic in Malaysia.

**Methods:**

An online questionnaire was distributed randomly to 1,050 HCWs from the Ministry of Health facilities in the Klang Valley who were involved directly in managing or screening COVID-19 cases from May to August 2020. The questionnaire was divided into five domains, which were concerns, impact on life and work, practice, perceived adequacy of preventive measures, and Revised Impact of Event Scale (IES-R). Logistic regression was used to identify sociodemographic predictors of the five domains.

**Results:**

A total of 907 respondents (86.4%) participated in this survey. Approximately half of the respondents had a low concern (50.5%), most of them had a good practice (85.1%), with 67.5% perceiving there were adequate preventive measures, and they perceived the outbreak had a low impact (92%) on their life and work. From the IES-R domain, 18.6% of respondents potentially suffered from post-traumatic stress disorder (PTSD).

**Conclusion:**

During the second wave of the COVID-19 outbreak in Malaysia, HCWs practiced high levels of precautions and preventive measures because they were aware of the risk of infection as an occupational hazard. With the adequate implementation of policy and control measures, the psychological wellbeing of the majority HCWs remained well and adequately supported.

## 1. Introduction

COVID-19 cases remain high worldwide, with approximately 6.5 million new cases reported in 7 days as of July 2022. The World Health Organization (WHO) warned of setbacks and new hurdles in changing viral variants and the need to gird for the epidemic to persist for a few more years (1). The first local case was detected in January 2020, demarcating the first wave of COVID-19 in Malaysia (2). In March 2020, Malaysia issued its first nationwide mobility control order due to a sharp rise in cases following the second wave of COVID-19 (3). Klang Valley, a two-federal territory and six-district metropolitan conglomeration on Peninsular Malaysia's west coast, had the most COVID-19 instances that accounted for nearly 40% of the national cases, and it was declared a Red Zone when the infection rose to more than 40 instances per day (2). The Ministry of Health Malaysia (MOH) has strategized the national COVID-19 prevention and control activities (i.e., contact tracking, close contact screening, handling, and monitoring suspected, verified, and under surveillance cases). Although all public and private healthcare facilities were deployed to tackle this epidemic, the public sector received a heavier workload and burden (4). Two public tertiary hospitals in the Klang Valley were designated as COVID-19 hospitals to exclusively manage active COVID-19 cases and provide critical care services for them. While close contact screening and surveillance activities were conducted by the primary care services involving HCWs from district health offices and health clinics.

During disease outbreaks, HCWs being the first responders are at greater risk of being exposed to biological hazards and contracting the disease. A meta-analysis of studies conducted in China, the United States, and Italy reported that more than 10% of all patients with COVID-19 were HCWs (5). In Malaysia, the incidence risk ratio of HCWs acquiring COVID-19 was nearly three times higher than the general population (6). Therefore, the risk of contracting COVID-19 infection from their workplace and the possibility of extending the risk to their family and close acquaintances were the most frequent concerns among HCWs (7, 8). In addition, the sociodemographic and occupational characteristics of the healthcare workers (HCWs) played a role in their level of concern. Higher age, post-graduate education, and working as a doctor were found to be associated with high concern during previous disease outbreaks (9). However, a previous study related to the severe acute respiratory syndrome (SARS) outbreak found that healthcare assistants were more concerned about their family's and others' health as compared to doctors (10).

With regard to COVID-19's impact on HCWs' life, a study reported that more than 50% HCWs felt stigmatized in various life domains such as quality of life, social contacts, and self-esteem previous studies (11). Another study among nursing professionals found that only 12–24% of nurses had perceived a high impact of COVID-19 on their life and family members. Nevertheless, their perceived impacts on work were reported to be slightly higher at 40–46% (8). The perceived impact of COVID-19 to work could be differed by job category. A study among HCWs in a teaching hospital showed that doctors had a higher perceived impact on working compared to nurses (12). Furthermore, another study related to the pandemic reported that HCWs' perceived impact on work was related to increased workload and the need to work overtime, especially in healthcare settings with high incidences of COVID-19 cases (13, 14).

In addition, good work practices toward COVID-19 and compliance with infection prevention control (IPC) were reinforced as key considerations for occupational safety and health (15). Earlier studies among HCWs showed good practices, and IPC compliance varied between 22 and 65% (16–18). There have been increased practices since the pandemic. However, more training sessions were needed on using personal protective equipment (PPE) and case management, including treatment (19, 20). Therefore, a systematic review was done on 20 studies that reported a higher median (78.8%) for good practices among HCWs associated with the type of profession, experience, age, level of education, use of personnel protective equipment, and gender (21).

Correspondingly, the level of preparedness among HCWs is crucial in building an appropriate response to the COVID-19 pandemic. These include strategic planning by providing support and education, offering prompt and authoritative information, and easing anxiety before an outbreak. A study of HCWs following the Avian Influenza pandemic in Singapore showed that most of them felt prepared regarding the availability of an informed workplace preparedness plan and regular infection control activities and the influenza vaccination program provided by the employer (14). HCWs in healthcare facilities that admit and actively manage confirmed cases of COVID-19 are at risk of contracting and transmitting the disease to their family members and others. Hence, compliance with the guidelines and policies on infection prevention control and occupational safety and health is essential to ensure their preparedness and protection from physical and psychological health risks.

Apart from concerns, perceived impact, practices, and preventive measures, there were also psychosocial impacts from long working hours leading to distress, fatigue, and occupational burnout (15). In addition to the increased workload during the screening and management of patients, the requirement to wear full PPE may have contributed to a stressful work environment that could impact the staff's mental health. A previous study in Canada concluded that HCWs who worked in hospitals treating SARS cases were prone to experience burnout, psychological distress, and post-traumatic stress compared to those who worked in the hospital with no SARS-related cases (22). A study conducted in a tertiary hospital in Taiwan reported similar findings that HCWs who treated patients in emergency settings during the SARS outbreak developed more severe post-traumatic stress disorder (PTSD) symptoms than staff in the psychiatric department (23). Many studies have found that HCWs directly exposed to patients with COVID-19 in their clinical settings were associated with a high risk of PTSD symptoms and other psychological disorders during the pandemic (24–26).

Given the scarcity of local evidence, it is crucial to assess the psychological health risk among HCWs who have been involved in COVID-19 management in Malaysia since the pandemic began. While previous studies addressed the psychological outcomes among HCWs within hospital settings, our study looked into exposures from different job categories and healthcare settings (i.e., hospital, clinic, and health office). This study will provide information on the current HCWs' situation during the outbreak and serve as a reference for monitoring the preparedness and psychological aspects of HCWs in the event of a disease outbreak in a developing country. The exposure of HCWs to COVID-19 infection at workplace may increase their concerns, impact their personal and professional lives, perceive good practices and adequate preventive measures, and impact their mental health. Certain sociodemographic (i.e., age, ethnicity, and family characteristics) and occupational (i.e., nature of work, job category, and workplace settings) factors may influence HCWs' perception and psychological outcomes during this pandemic. Thus, this study aimed to measure concerns, practices, perceived impact, preventive measures, and stress among HCWs as well as describe the associated sociodemographic and occupational factors.

## 2. Methods

### 2.1. Study design and area

This is a cross-sectional study conducted from May to August 2020 in three different settings of public healthcare facilities under the MOH in Klang Valley. The Klang Valley area, also known as Greater Kuala Lumpur, covers the Federal Territories of Kuala Lumpur and Putrajaya, as well as six districts in Selangor State, including Petaling, Klang, Gombak, Hulu Langat, Sepang, and Kuala Langat. This study was conducted in 103 public healthcare facilities that manage COVID-19 cases, consisting of 10 hospitals, 13 district health offices, and 80 health clinics.

### 2.2. Sampling method

The list of HCWs involved with COVID-19-related activities during the second wave of the COVID-19 pandemic was obtained from the Occupational Health Unit of each state health department in Klang Valley. The inclusion criteria for respondents in this study were HCWs who had a risk of direct or indirect exposure to COVID-19 while handling and managing patients with COVID-19 for at least 7 days during the study period. The term direct exposure used in this study referred to the case definition on the MOH guidelines for COVID-19 management in Malaysia, which is defined as a person who has exposure to a probable or confirmed case within 1 m and for at least 15 min (27). While for exclusion criteria, HCWs in government healthcare facilities outside Klang Valley and other government healthcare facilities, which are not involved in screening and managing COVID-19 cases, were excluded.

A total of 6,736 HCWs fulfilled the criteria and were eligible to participate in this study. The COVID-19-related activities in healthcare facilities vary according to the work's nature. HCWs in hospitals who were mostly working in the emergency department, intensive care unit (ICU), and wards were exposed to COVID-19 during the admission process, which persisted throughout patients' stay until discharge. Since the MOH designated only a few hospitals for managing in-patient COVID-19 cases at the time of this study, all HCWs in the specified departments were included. Whereas, HCWs from district health offices were involved in various public health and clinical work such as contact tracing, patient screening, triaging, and conducting field investigations on COVID-19 cases and clusters. They could be exposed during sample swabbing activities, transporting, and transferring confirmed cases to designated hospitals. These exposures were apparent during managing large COVID-19 clusters that required mass screening.

In this study, the questionnaire was self-administered using an online survey tool in the form of bilingual (English and Malay language). Each selected respondent will be given an ID number to ensure anonymity. The respondent can answer the questionnaires online *via* computers or mobile phones. Participants will take ~10–15 min to complete the questionnaire. The questionnaire will be distributed to selected healthcare facilities. Participation will be voluntary and anonymous. The consent form and research information have been included in the online questionnaire. The respondent must select “agree and continue” to consent to the study. Once all questionnaires had been filled online, automatically, the data were recorded in a spreadsheet in an analyzable format and allowed for tabulation and graphical representations.

All 6,736 eligible HCWs from 103 healthcare facilities were coded and listed in Microsoft Excel. A random number was then generated to select 1,050 participants, and the invitation to participate in this study was emailed to them. We received 923 responses, of which 907 respondents had completed the questionnaire, and only 16 declined to participate.

### 2.3. Study tool

The questionnaire was adapted from previous studies on SARS, avian flu, and Middle East respiratory syndrome (MERS) outbreaks (9, 14, 28). The questionnaire was structured into two parts: The first part collected sociodemographic data on respondents, occupations, and family history. The next part consisted of 54 items that were divided further into five domains: (i) concerns about their involvement in managing the pandemic, (ii) practices of control measures in workplace settings, (iii) perception regarding the adequacy of implemented preventive measures, (iv) impact of COVID-19 to personal and professional lives, and (v) psychological impact of COVID-19 of stress (PTSD).

The responses in the first four domains on concerns, the practice of control measures, perceived preventive measures, and the COVID-19 impact were assessed using a Likert scale with 1- or 4-point ordinal points (strongly disagree = 1, disagree = 2, agree = 3, and strongly agree = 4). All points from each domain were summed up and then classified into two categories based on the total point percentage, which include low or high concern and impact, poor or good practice, and inadequate or adequate preventive measures. The percentage score of 75% and above was used as a cutoff between those two categories. The cutoff point has been chosen for capturing more samples and giving meaningful results. These first four domains were validated at the onset of the study involving 220 samples. Cronbach's alpha coefficients were between 0.740 and 0.917 for all the domains (29). The details of each of the first domains are described later.

- The first domain was about concerns by HCWs regarding COVID-19. The questionnaire included 14 work-related items (nine items) and non–work-related items (five items). It was used to measure the perceived risk of contracting COVID-19 disease through their exposure at the workplace, and the risk of transmitting it to people close to them.
- The second domain focused on infection control prevention practices among HCWs. The questionnaire included 15 items, including availability and adherence to the infection control protocols, and compliance with personal protection equipment (PPE).
- The third domain was about the implementation of preventive measures at the workplace. The questionnaire included eight items on the provisions to protect HCWs through infection control measures, implementation of clear policies and protocols of infection control at the workplace, and about staff adherence.
- The fourth domain measures the impact of COVID-19 on the HCWs' personal (three items) and works life (four items), including perceived social stigmatization and issues at the workplace, such as conflict, stress, and high workload.

The last domain was on the psychological impact of COVID-19 using 10 items from the Revised Impact of Event Scale (IES-R). The IES-R was chosen to assess psychological impact by reviewing the degree of distress among respondents (30). The rationale for choosing IES-R was due to short, self-administered questionnaires that can be answered by those individuals exposed to traumatic events regardless of their health status. Moreover, the criteria delineated in the Diagnostic and Statistical Manual of Mental Disorders, Fourth Edition (DSM-IV) to assess PTSD were incorporated in the questions (31). It reviewed the intrusive and avoidance symptoms at least 7 days after HCWs were exposed to COVID-19 cases, and the components are interpreted as a total score to be used for preliminary diagnosis of PTSD. We used 10 items questionnaire to assess four intrusion items, five avoidance items, and one hyperarousal item (32). This last domain was assessed according to the original scale of 0- or 5-point ordinal points that range from “not at all” to “extremely” (33). The interpretation of 10 items questionnaire for a total score of 15 and more is categorized as “more likely” to have PTSD. Those who scored < 15 were grouped into “less likely” to have PTSD. This IES-R domain was already validated for both languages (29, 30, 32). The scale of having internal consistency can be implied when Cronbach's alpha is higher than 0.7. Cronbach's alpha for the three subdomains ranged from 0.87 to 0.92 (31).

### 2.4. Study analysis

The analysis was carried out using Statistical Package for the Social Science software (SPSS) version 24.0. Categorical data were described by frequency and percentage distribution, while continuous data were described using mean and standard deviation (SD). Logistic regression analysis was performed between each sociodemographic variable with all five domains to identify the covariates for the best-fit model. Variables with a *p* < 0.25 were selected and included in the final model (34). A multivariate analysis was conducted using binomial logistic regression with the selected variables to calculate the adjusted odds ratio (AOR) with a 95% confidence interval (95%CI). The dependent variables chosen in logistic regression were high concern and impact, good practice, adequate preventive measures, and more likely to have PTSD coded as 1. A *p* < 0.05 was considered statistically significant.

### 2.5. Ethics consideration

Ethics approval is required before the commencement of research within the MOH's healthcare facilities and involving HCWs. The Medical Research and Ethics Committee (MREC) approved the ethical study under reference number KKM/NIHSEC/P20-715(6). All participants were anonymous, with no personal identifiers in any part of the analysis or report. Respondents who consented could proceed to the next part of the online survey and were required to answer all questions before submission. Participants who refused were excluded from further analysis.

## 3. Results

**Figure 1:**
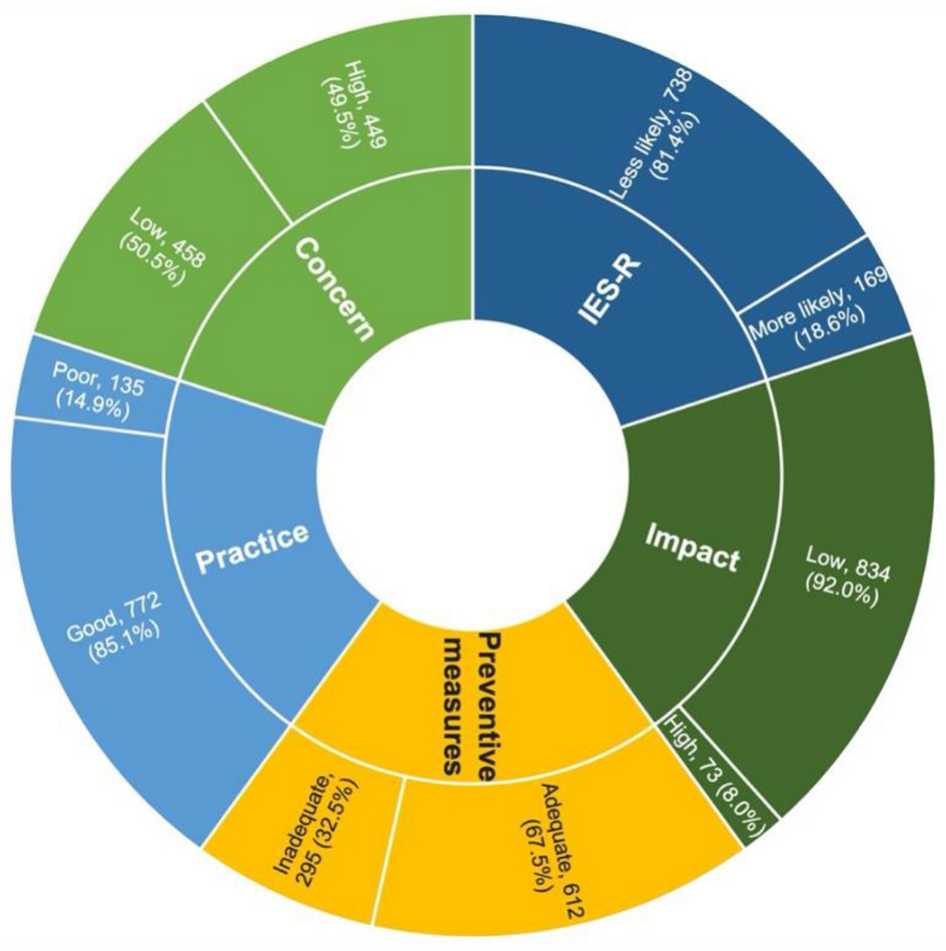
Percentage of concerns, practice, adequacy of preventive measures, impact, and IES-R score among HCWs in Klang Valley.

A total of 1,050 HCWs were randomly selected to participate in this online survey, and the response rate was 86.4%, involving 907 respondents. As shown in Table 1, the majority of respondents were women (62.6%), Malay ethnicity (80.7%), and married (73.2%) with children (64.8%). Most respondents were between 30 and 39 years old (51.8%) with a mean ± SD age of 33.71 ± SD 6.684 years. In terms of the workplace, more than half of the respondents were from health clinics (56.1%), followed by hospitals (27.9%) and district health offices (16%). Approximately one-third of respondents were allied health staff (33.6%) with a duration of work of more than 5 years (67.1%). In addition, most of the respondents had direct contact with COVID-19 confirmed cases at their workplace (86.1%), with more than one-quarter of them having frequent contact of more than 3 days per week (25.6%). Most of the respondents have low concern (50.5%), good work practice (85.1%), perceived adequate preventive measures (67.5%), and low impact (92%), as shown in Figure 1. For the IES-R domain, the majority (81.4%) of respondents were less likely to suffer from PTSD. The details of respondents' responses according to each item for concerns, practice, preventive measures, impact, and IES-S are tabulated in Supplementary material.

**Table 1:** Distribution of respondents based on the demographical and occupational characteristics.

**Variables**	***n* (907)**	**%**
**Gender**		
Male	339	37.4
Female	568	62.6
**Age**		
Below 30 years old	292	32.2
30–39 years old	470	51.8
40 and above	145	16.0
**Ethnicity**		
Malay	732	80.7
Chinese	50	5.5
Indian	85	9.4
Others	40	4.4
**Marital status**		
Single	234	25.8
Ever married	673	74.2
**No. of child**		
No child	319	35.2
1–3 children	506	55.8
More than 3 children	82	9.0
**Healthcare facilities**		
Hospital	253	27.9
Health clinic	509	56.1
District health office	145	16.0
**Profession**		
Doctors	297	32.7
Nurses	258	28.4
Allied health staffs	305	33.6
Others	47	5.2
**Years of service**		
< 3 years	111	12.2
3–5 years	187	20.6
6–10 years	325	35.8
>10 years	284	31.3
**Direct contact frequency**		
No direct contact	191	21.1
6–7 days a week	209	23.0
3–5 days a week	275	30.3
>3 days a week	232	25.6

Table 2 shows that five out of nine variables had a *p* < 0.25 based on univariate analysis: ethnicity, number of children, facility type, profession, and frequency of direct contact. Based on ethnicity, Indian respondents were 2.4 times more likely to score higher practice than Malay respondents (95% CI 1.03–5.70, *p* < 0.05). The odds for Chinese and others ethnicity for more likely PTSD were two times higher than Malay and Indian. Respondents with more than three children had two times higher odds of perceived adequate preventive measures than respondents with no child (95% CI 1.16–3.70, *p* < 0.05). Based on facility type, respondents from health clinics had 30% more odds of perceived adequate preventive measures than respondents from hospitals (95% CI 0.97–1.83, *p* < 0.05). Respondents from district health offices had two times the odds of having “more likely PTSD” as respondents from the hospital (95% CI 1.13–3.03, *p* < 0.05). Based on profession, nurses and allied health staff had 2- and 1.6-times higher odds for high perceived adequate preventive measures compared to doctors (95% CI 1.38–2.85, 95% CI 1.15–2.26, *p* < 0.05). The odds of “high concern” were much higher with increased frequency of direct contact. For respondents with frequent direct contact with patients with COVID-19 (6–7 days a week), the odds of high concern, high work practice, and perceived adequate preventive measures were nearly 2–3 times higher compared to respondents with no direct contact.

**Table 2:** Sociodemographic and occupational factors associated with concern, practice, preventive measure, impact, and IES-R domain.

**Variables**	**Concern**		**Practice**		**Preventive measure**		**Impact**		**IES-R**	
	**OR (95% CI)**	**AOR (95% CI)**	**OR (95% CI)**	**AOR (95% CI)**	**OR (95% CI)**	**AOR (95% CI)**	**OR (95% CI)**	**AOR (95% CI)**	**OR (95% CI)**	**AOR (95% CI)**
**Ethnicity**										
Malay	1	1	1	1	1	1	1	1	1	1
Chinese	1.04 (0.58–1.84)	1.23 (0.66–2.28)	1.35 (0.56–3.25)	1.09 (0.43–2.78)	0.43^*^ (0.24–0.77)	0.58 (0.31–1.07)	1.86 (0.80–4.31)	1.63 (0.64–4.10)	2.02^*^ (1.07–3.81)	2.33^*^ (1.16–4.69)
Indian	0.52^*^ (0.33–0.83)	0.58^*^ (0.35–0.96)	2.43^*^ (1.03–5.70)	2.40 (0.99–5.85)	0.72 (0.45–1.14)	0.97 (0.59–1.60)	0.42 (0.13–1.36)	0.39 (0.11–1.32)	0.93 (0.51–1.70)	1.14 (0.59–2.18)
Others	0.87 (0.46–1.64)	1.04 (0.53–2.03)	0.64 (0.29–1.37)	0.72 (0.32–1.62)	0.59 (0.31–1.12)	0.59 (0.30–1.15)	1.27 (0.44–3.68)	1.43 (0.47–4.32)	2.02^*^ (1.00–4.08)	1.62 (0.78–3.39)
**No. of children**										
No child	1	1	1	1	1	1	1	1	1	1
1–3 children	1.23 (0.93–1.62)	1.29 (0.95–1.76)	0.76 (0.51–1.14)	0.82 (0.53–1.26)	1.11 (0.83–1.49)	0.94 (0.68–1.30)	1.02 (0.61–1.70)	0.92 (0.52–1.62)	0.75 (0.53–1.06)	0.77 (0.52–1.12)
>3 children	0.99 (0.61–1.60)	0.96 (0.57–1.62)	1.23 (0.57–2.64)	1.23 (0.55–2.73)	2.07^*^ (1.16–3.70)	1.59 (0.87–2.92)	0.73 (0.27–1.97)	0.62 (0.22–1.74)	0.43^*^ (0.21–0.90)	0.44^*^ (0.20–0.95)
**Facility type**										
Hospital	1	1	1	1	1	1	1	1	1	1
Health clinic	1.04 (0.77–1.40)	1.22 (0.87–1.71)	0.71 (0.45–1.14)	0.83 (0.50–1.38)	1.33 (0.97–1.83)	1.39 (0.98–1.98)	1.41 (0.79–2.51)	1.68 (0.89–3.19)	1.05 (0.70–1.57)	1.11 (0.71–1.73)
District health office	0.61^*^ (0.39–0.92)	0.65 (0.42–1.01)	0.38^*^ (0.22–0.65)	0.45^*^ (0.25–0.81)	1.09 (0.71–1.67)	1.17 (0.74–1.85)	0.92 (0.39–2.12)	1.21 (0.50–2.93)	1.85^*^ (1.13–3.03)	2.13^*^ (1.25–3.64)
**Profession**										
Doctors	1	1	1	1	1	1	1	1	1	1
Nurses	1.38 (0.99–1.93)	1.16 (0.81–1.68)	0.97 (0.59–1.60)	1.08 (0.63–1.85)	1.99^*^ (1.38–2.85)	1.81^*^ (1.22–2.68)	1.27 (0.72–2.25)	1.18 (0.63–2.21)	1.38 (0.89–2.12)	1.84^*^ (1.13–2.99)
Allied health staffs	1.27 (0.92–1.76)	1.13 (0.79–1.62)	0.68 (0.43–1.07)	0.81 (0.49–1.33)	1.61^*^ (1.15–2.26)	1.48^*^ (1.02–2.15)	0.64 (0.34–1.22)	0.56 (0.28–1.12)	1.36 (0.89–2.06)	1.42 (0.89–2.29)
Others	1.07 (0.58–1.98)	0.83 (0.43–1.62)	0.42^*^ (0.19–0.87)	0.51 (0.23–1.13)	1.64 (0.84–3.20)	1.43 (0.71–2.86)	1.01 (0.34–3.05)	0.86 (0.27–2.76)	0.93 (0.39–2.20)	1.13 (0.46–2.79)
**Direct contact frequency**										
None	1	1	1	1	1	1	1	1	1	1
6–7 days a week	2.78^*^ (1.85–4.16)	3.04^*^ (1.98–4.68)	1.75^*^ (1.02–3.01)	1.84^*^ (1.04–3.28)	1.68^*^ (1.11–2.55)	1.77^*^ (1.14–2.76)	1.92 (0.87–4.20)	2.45^*^ (1.08–5.57)	0.99 (0.59–1.68)	1.01 (0.59–1.75)
3–5 days a week	1.71^*^ (1.18–2.49)	1.69 (1.15–2.49)	1.46 (0.89–2.38)	1.44 (0.87–2.38)	1.48^*^ (1.00–2.17)	1.54^*^ (1.03–2.29)	1.73 (0.81–3.71)	1.87 (0.86–4.06)	1.28 (0.79–2.06)	1.38 (0.84–2.25)
>3 days a week	1.39 (0.94–2.05)	1.41 (0.94–2.12)	1.61 (0.96–2.71)	1.75 (1.02–3.00)	1.38 (0.93–2.06)	1.47 (0.97–2.23)	1.62 (0.73–3.56)	1.67 (0.74–3.75)	1.06 (0.64–1.75)	1.09 (0.65–1.83)

High concern, good practice, adequate preventive measures, high impact, and more likely to have PTSD (IES-R) coded as 1. OR, odd ratio; AOR, adjusted odd ratio; ^*^indicates a significant p < 0.05.

The binomial logistic regression analysis between all domains with sociodemographic variables found that ethnicity, type of healthcare facilities, professions, years of service, and frequency of direct contact with patients with COVID-19 were fitted in the final model, as shown in Table 2. The Hosmer–Lemeshow test was not significant for all the models (*p* > 0.05), and the classification table showed that the overall model was correctly classified with a percentage of more than 70%. From the analysis, the respondents with a direct contact frequency of 6–7 days a week had higher odds of having higher concern, good practice, perceived adequate preventive measures, and higher impact than respondents with a direct contact frequency of < 6 days a week.

From the IES-R domain, the respondents with more than three children had 56% fewer odds of having PTSD. Based on occupational factors, nurses are two times more likely to suffer from PTSD compared to other professions (95% CI 1.13–2.99, *p* < 0.05). Meanwhile, respondents from the district health office were two times more likely to suffer from PTSD than hospital HCWs (95% CI 1.25–3.64, *p* < 0.05). The odds of using PPE were higher among nurses and allied health staff (1.8 and 1.5 times, respectively), than among doctors. Chinese respondents had two times the odds of likely suffering from PTSD compared to Malay respondents (95% CI 1.16–4.69, *p* < 0.05). Respondents with direct contact with patients with COVID-19 (6–7 days a week) had three times the odds for high concern and 2.5 times the odds for high impact compared to the respondent with no direct contact (95% CI 1.98–4.68, 95% CI 1.08–5.57, *p* < 0.05).

## 4. Discussion

The COVID-19 pandemic has affected and greatly burdened the healthcare system, particularly the frontline workers. HCWs were the most affected, as they faced emerging unknown infectious diseases. At the same time, they carried on the duty to deliver health services and treatment to others. This study was done when the burden of new COVID-19 cases in Malaysia started to climb in Klang Valley, and the proportion of HCWs became less compared to patients. This study found that approximately 50% of respondents have a low concern, good practice (85.1%), with perceived adequate preventive measures (67.5%), and perceived low impact (92%) on their life and work from managing the COVID-19 pandemic. However, < 20% of the respondents were more likely to suffer from PTSD. Our study further indicated that the frequency of direct contact with patients with COVID-19 influences the odds of having high concern, high work practice, and perceived adequate preventive measures. This result might highlight the preparedness and resilience of the HCWs in facing the pandemic.

During the COVID-19 pandemic, HCWs were at risk of getting infected while working due to constant exposure (35). Their major involvement with screening and providing treatment at all levels of healthcare institutions puts them at risk of contracting the disease. MOH Malaysia was very proactive and issued frequently updated guidelines on managing COVID-19 cases and infection protection control (IPC) measures. Therefore, even before the study was conducted, HCWs were diligent in preventive measures as they were trained and updated with the latest guidelines. Nearly all (96.7%) respondents agreed that the policies and protocols implemented were timely and easy to follow. On the other hand, it was suggested that lack of proper PPE training would increase the risk of HCWs exposure in the workplace (36). Our study showed more that 95% agreed that there were adequate training for PPE applications and supplies. This finding is varied in other countries. For example, in Australia, most of their emergency clinicians (77.6–86.4%) reported receiving specific training and education on COVID-19, including PPE usage (37). While in North Central Ethiopia, only half (49.8%) of their healthcare providers were prepared for the COVID-19 pandemic (38).

Almost half of the respondents had great concerns about the risk of infection and mostly had good practices on wearing full PPE and compliance with SOPs. Hospitals and health clinics had higher concerns compared to those working in the district health office, as their scope of work in these facilities involved direct close contact with patients and constant exposure throughout their shifts. HCWs involved with clinical work in hospitals and health clinics during the pandemic had a higher prevalence of stress, fear, and anxiety compared to HCWs in non-clinical settings (39). More than 85% of our respondents showed concerns about the possibility of transmitting the infection to their family members and friends due to the nature of their work as compared to other studies (39, 40). The frequency of direct contact with patients with COVID-19 had shown to be the predictor for higher concern and impact. This is most likely because they were at a higher risk of infection than those with less contact with patients with COVID-19. However, the time of exposure influences the risk of infection. If exposure to patients with COVID-19 occurred on day 2 or 3, the risk of contracting the disease is higher (41). Similar findings were also shown in other infectious disease outbreaks, of which daily contact and exposure were more likely to have a higher psychological impact and concern (9, 28, 39). Another study also reported that the degree of contact with COVID-19 cases was directly related to mental health illness (42).

Healthcare workers working at the district health office had significantly less practice than others. This is most likely because they did not directly examine or attend to the patient. Instead, they are practically more involved in community-based surveillance (43). Furthermore, HCWs working with the district health office are less concerned than respondents from the other two types of healthcare facilities. However, their IES-R scores were high, indicating that they were more likely to suffer from PTSD. This could be due to an increased workload due to a lack of human resources. To address this issue, the government directed that healthcare personnel be deployed to various healthcare facilities facing a manpower shortage. This is echoed by data published by the National Institutes of Health Malaysia (NIH), a total of 128 personnel, primarily medical officers from health institutes, have been mobilized to various healthcare facilities. During the study period, 44 health personnel were mobilized to the district health office (34%) and 12 to hospitals in the Klang Valley (44).

In this study, staff nurses and allied health personnel were significantly more likely than doctors to implement preventive measures. Although, according to the qualitative research conducted by Efstathiou et al. among nurses, factors such as the high risk of infection and vulnerability to disease were the reasons for preventive measure implementation. The benefits of taking preventative measures make them feel calm while attending to patients, according to the same study (45). This is echoed by a study in Palestine, where almost 92% of the nurses used preventive measures while handling patients with COVID-19 (46). However, this study found that nurses are more likely to develop PTSD. These findings can be supported by the high level of stress that they encountered during the pandemic. PTSD is caused by traumatic events and can further lead to other psychological disorders. HCWs are responsible in taking care of COVID-19 patient with longer contact time, thus, increase their risk of infection (26). This might lead as a contributing factor for them being more likely to adhere to preventive measures, but at the same time, becoming a burden on their mental health (24). Most staff nurses in Malaysia were female. It has been supported that female participants were at high risk of developing mental disorders in most infectious disease outbreak studies (47–49).

Most HCWs in the study also showed a low prevalence of impact despite increased workload and additional hours worked during the pandemic. The likely reason for this could be the adequate physical and emotional support they received. In the area of perceived adequate preventive measures, most of them agreed (91.5%) that they received emotional support when they needed help. Regarding family support, the study found that those with more children were 56% less likely to have PTSD. These findings suggest that those with more family members have better mental wellbeing. Some healthcare workers may avoid the community or family when working in COVID-19 facilities. Therefore, connecting with their relatives or trusted people can strengthen their moral support (50). However, some studies have found that healthcare workers' fear of infection and possible infection of their family members may contribute to psychological distress associated with a pandemic (22, 51, 52). Therefore, further evaluation is needed to explore more factors that might contribute to the result.

Among the study's limitations was that the respondents from different backgrounds in healthcare settings contributed to this study. They have different roles and job tasks that might have different types of exposure to COVID-19. HCWs in hospitals were aware of their exposure as their settings already have proper planning and preparedness such as proper PPE, isolation rooms, and proper triage settings for any infectious disease and COVID-19. While in health clinics and district health offices, they have unknown exposures and need to be always cautious as their settings need better equipment and proper plan like the hospital does. These might affect their overall exposure to COVID-19 and influence the result of the five domains quantified in this study. On the other hand, the HCWs had time constraints to participate and complete the survey. However, most of the respondents at that time were actively involved and occupied in managing COVID-19 cases. Response to the study was good among HCWs from hospitals and health clinics but relatively poor (41.4%) among HCWs working in the district health office. This could be attributed to the heavy workload at healthcare facilities during the peak of the second wave of COVID-19, where the management of COVID-19 from the screening process, contact tracing, and swab sampling to transporting patients to hospitals was taking place. As mentioned earlier, the shortage of staff from the district health office might have led to poor response due to limitations in answering the questionnaire. Apart from that, this study was only conducted in the Klang Valley area and might not represent the whole of Malaysia. However, most of the COVID-19 cases were detected and admitted to Klang Valley healthcare facilities during the data collection.

## 5. Conclusion

In conclusion, the majority HCWs had good work practices and perceived adequate preventive measures as they were aware of their exposure and risk of getting infected. Furthermore, our study found that HCWs with frequent direct contact with the patient were more likely to have high concerns and impacts on their personal and social life when managing COVID-19 cases. However, their psychosocial wellbeing remains well-supported as no associations were found with PTSD. Therefore, worksite health promotion programs to address COVID-19 concerns should focus on HCWs with higher COVID-19 exposure risks. With the implementation of policy and control measures, the psychological wellbeing of HCWs remains supported, and the prevalence of mental health illness can be reduced.

## Data availability statement

The raw data supporting the conclusions of this article will be made available by the authors, without undue reservation.

## Ethics statement

This study involving human participants was reviewed and approved by the Medical Research and Ethics Committee (MREC), Ministry of Health Malaysia [KKM/NIHSEC/ P20-715 (7)]. The patients/participants provided their written informed consent to participate in this study.

## Author contributions

MAP, RI, NM, RMR, SR, SSAT, ASAS, and NR participated in the data acquisition. MAP, YCL, and RI analyzed the data and interpreted the results. MAP, RI, RMR, YCL, and RS wrote the manuscript. All the authors were involved in the conception and design of the research, critically revised the manuscript, and read and agreed to the published version of the manuscript.

## References

[1] World Health Organization. Weekly Epidemiological Update on COVID-19 - 23 March 2021. Geneva: World Health Organization (2021). Available online at: https://www.who.int/publications/m/item/weekly-epidemiological-update-on-covid-19-−23-march-2021 (accessed March 29, 2021).

[2] Ministry of Health Malaysia. Situasi Semasa Jangkitan Penyakit Coronavirus 2019 (Covid-19) Di Malaysia 13 Mac 2020. Putrajaya: Kementerian Kesihatan Malaysia (KKM) (2020). Available online at: https://www.moh.gov.my/index.php/database_stores/attach_download/337/1353 (accessed August 14, 2020).

[3] Shah AUM Safri SNA Thevadas R Noordin NK Rahman AA Sekawi Z. COVID-19 outbreak in Malaysia: actions taken by the Malaysian government. Int J Infect Dis. (2020) 97:108–16. 10.1016/j.ijid.2020.05.093 32497808 PMC7264933

[4] Tan CS Lokman S Rao Y Kok SH Ming LC. Public and private sectors collective response to combat COVID-19 in Malaysia. J Pharm Policy Prac. (2021) 14:40. 10.1186/s40545-021-00322-x 33941265 PMC8091142

[5] Sahu AK Amrithanand VT Mathew R Aggarwal P Nayer J Bhoi S. COVID-19 in health care workers – a systematic review and meta-analysis. Am J Emer Med. (2020) 38:1727–31. 10.1016/j.ajem.2020.05.113 32738467 PMC7837172

[6] Harith AA Ab Gani MH Griffiths R Abdul Hadi A Abu Bakar NA Myers J. Incidence, prevalence, and sources of COVID-19 infection among healthcare workers in hospitals in Malaysia. Int J Environ Res Public Health. (2022) 19:12485. 10.3390/ijerph191912485 36231783 PMC9564780

[7] Sahashi Y Endo H Sugimoto T Nabeta T Nishizaki K Kikuchi A. Worries and concerns among healthcare workers during the coronavirus 2019 pandemic: a web-based cross-sectional survey. Hum Soc Sci Commun. (2021) 8:41. 10.1057/s41599-021-00716-x

[8] Galletta M Piras I Finco G Meloni F D'Aloja E Contu P. Worries, preparedness, and perceived impact of covid-19 pandemic on nurses' mental health. Front Public Health. (2021) 9:566700. 10.3389/fpubh.2021.566700 34123979 PMC8187773

[9] Abolfotouh MA AlQarni AA Al-Ghamdi SM Salam M Al-Assiri MH Balkhy HH. An assessment of the level of concern among hospital-based health-care workers regarding MERS outbreaks in Saudi Arabia. BMC Infect Dis. (2017) 17:4. 10.1186/s12879-016-2096-8 28049440 PMC5210292

[10] Wong TW Yau JKY Chan CLW Kwong RSY Ho SMY Lau CC. The psychological impact of severe acute respiratory syndrome outbreak on healthcare workers in emergency departments and how they cope. Euro J Emerg Med. (2005) 12:13–8. 10.1097/00063110-200502000-00005 15674079

[11] Radhakrishnan RV Jain M Mohanty CR Jacob J Shetty AP Stephen S. The perceived social stigma, self-esteem, and its determinants among the health care professionals working in India during COVID 19 pandemic. Med J Armed Forces India. (2021) 77:S450–8. 10.1016/j.mjafi.2021.01.017 34393330 PMC8346812

[12] Saurabh K Ranjan S. Preparedness, perceived impact and concerns of health care workers in a teaching hospital during coronavirus disease 2019 (COVID-19). J Family Med Prim Care. (2020) 9:4247–51. 10.4103/jfmpc.jfmpc_799_20 33110840 PMC7586576

[13] Carmassi C Dell'Oste V Barberi FM Bertelloni CA Pedrinelli V Dell'Osso L. Mental health symptoms among general practitioners facing the acute phase of the COVID-19 pandemic: detecting different reaction groups. Int J Environ Res Public Health. (2022) 19:4007. 10.3390/ijerph19074007 35409690 PMC8998411

[14] Wong TY Koh GC Cheong SK Lee HY Fong YT Sundram M. Concerns, perceived impact and preparedness in an avian influenza pandemic–a comparative study between healthcare workers in primary and tertiary care. Ann Acad Med Singap. (2008) 37:96–102. 18327343

[15] WHO. Coronavirus Disease (COVID-19) Outbreak: Rights, Roles and Responsibilities of Health Workers, Including Key Considerations for Occupational Safety and Health: Interim Guidance, 19 March 2020. World Health Organization (2020). Available online at: https://apps.who.int/iris/handle/10665/331510 (accessed January 22, 2022).

[16] Mohamad N Pahrol MA Shaharudin R Md Yazin NKR Osman Y Toha HR. Compliance to infection prevention and control practices among healthcare workers during COVID-19 pandemic in Malaysia. Front Public Health. (2022) 10:878396. 10.3389/fpubh.2022.878396 35923958 PMC9340217

[17] Bahegwa RP Hussein AK Kishimba R Hokororo J German C Ngowi R. Factors affecting compliance with infection prevention and control standard precautions among healthcare workers in Songwe region, Tanzania. Infect Prev Pract. (2022) 4:100236. 10.1016/j.infpip.2022.100236 36052313 PMC9424571

[18] Patwary MM Hossain MdR Sultana R Dazhamyar AR Parsa AD Kabir R. Knowledge, attitudes and practices of healthcare professionals toward the novel coronavirus during the early stage of COVID-19 in a lower-and-middle income country, Bangladesh. Front Public Health. (2022) 10:988063. 10.3389/fpubh.2022.988063 36187704 PMC9523603

[19] Mbamalu O Surendran S Nampoothiri V Bonaconsa C Edathadathil F Zhu N. Survey of healthcare worker perceptions of changes in infection control and antimicrobial stewardship practices in India and South Africa during the COVID-19 pandemic. IJID Regions. (2022) 6:90–8. 10.1016/j.ijregi.2022.11.010 36466212 PMC9703863

[20] Mantes J Pandya-Orozco BP. Implementing infection prevention and control (IPC) practices including COVID-19 mitigation strategies in a skilled nursing facility. Am J Infect Cont. (2022) 50:S17. 10.1016/j.ajic.2022.03.091

[21] Tegegne GT Kefale B Engidaw MT Degu A Tesfa D Ewunetei A. Knowledge, attitude, and practice of healthcare providers toward novel coronavirus 19 during the first months of the pandemic: a systematic review. Front Public Health. (2021) 9:606666. 10.3389/fpubh.2021.606666 34249826 PMC8267791

[22] Maunder R Lancee W Balderson K Bennett J Borgundvaag B Evans S. Long-term psychological and occupational effects of providing hospital healthcare during SARS outbreak. Emerg Infect Dis. (2006) 12:1924–32. 10.3201/eid1212.060584 17326946 PMC3291360

[23] Lin C Peng Y Wu Y Chang J Chan C Yang D. The psychological effect of severe acute respiratory syndrome on emergency department staff. Emerg Med J. (2007) 24:12–7. 10.1136/emj.2006.035089 17183035 PMC2658141

[24] Buselli R Corsi M Baldanzi S Chiumiento M Del Lupo E Dell'Oste V. Professional quality of life and mental health outcomes among health care workers exposed to SARS-CoV-2 (Covid-19). Int J Environ Res Public Health. (2020) 17:6180. 10.3390/ijerph17176180 32858810 PMC7504107

[25] Carmassi C Pedrinelli V Dell'Oste V Bertelloni CA Cordone A Bouanani S. Work and social functioning in frontline healthcare workers during the covid-19 pandemic in Italy: role of acute post-traumatic stress, depressive and anxiety symptoms. Riv Psichiatr. (2021) 56:189–97. 10.1708/3654.36346 34310576

[26] Carmassi C Foghi C Dell'Oste V Cordone A Bertelloni CA Bui E. PTSD symptoms in healthcare workers facing the three coronavirus outbreaks: what can we expect after the COVID-19 pandemic. Psychiatry Res. (2020) 292:113312. 10.1016/j.psychres.2020.113312 32717711 PMC7370915

[27] Ministry of Health Malaysia. Guidelines COVID-19 Management in Malaysia No. 4/2020 (updated on 26 February 2020). Putrajaya: Ministry of Health Malaysia (2020).

[28] Koh D Lim MK Chia SE Ko SM Qian F Ng V. Risk perception and impact of severe acute respiratory syndrome (SARS) on work and personal lives of healthcare workers in Singapore: what can we learn? Med Care. (2005) 43:676–82. 10.1097/01.mlr.0000167181.36730.cc 15970782

[29] Ameerah Su'ad AS Muhammad Alfatih P Nurfatehar R Sakshaleni R Syahidiah SAT Rafiza S. Reliability and factor analyses of a questionnaire measuring concerns and perceptions of health care workers in Malaysia towards COVID-19 pandemic. IMRJ. (2022) 8.

[30] Weiss DS. The impact of event scale: revised. In:Wilson JP Tang CS, editors. Cross-Cultural Assessment of Psychological Trauma PTSD. Boston, MA: Springer (2007). p. 219–38. 10.1007/978-0-387-70990-1_10

[31] Sharif Nia H Kaur H Fomani FK Rahmatpour P Kaveh O Pahlevan Sharif S. Psychometric properties of the impact of events scale-revised (IES-R) among general Iranian population during the COVID-19 pandemic. Front Psychiatry. (2021) 12:692498. 10.3389/fpsyt.2021.692498 34408679 PMC8365034

[32] Norhayati MM, Aniza, AA. Psychometric Properties of the Malay version of Impact of Event Scale - Revised (IES-R). Undefined. (2014). Available online at: https://www.semanticscholar.org/paper/Psychometric-Properties-of-the-Malay-version-of-Mn-Aa/3033ec481b37a9bf3e3df677ddb63ce0db2b4fe3 (accessed June 23, 2022).

[33] Weiss DS Marmar CR. The impact of event scale—revised. In:Wilson JP Keane TM, editors. Assessing Psychological Trauma PTSD. New York, NY: The Guilford Press (1997). p. 399–411. 10.1037/t12199-000

[34] Bendel RB Afifi AA. Comparison of stopping rules in forward “stepwise” regression. J Am Stat Assoc. (1977) 72:46–53. 10.1080/01621459.1977.10479905

[35] Nguyen LH Drew DA Graham MS Joshi AD Guo C-G Ma W. Risk of COVID-19 among front-line health-care workers and the general community: a prospective cohort study. Lancet Public Health. (2020) 5:e475–83. 10.1101/2020.04.29.20084111 32745512 PMC7491202

[36] Puro V Nicastri E. SARS and the removal of personal protective equipment. CMAJ. (2004) 170:930. 10.1503/cmaj.1031700 15023907 PMC359412

[37] Li C Sotomayor-Castillo C Nahidi S Kuznetsov S Considine J Curtis K. Emergency clinicians' knowledge, preparedness and experiences of managing COVID-19 during the 2020 global pandemic in Australian healthcare settings. Austral Emerg Care. (2021) 24:186–96. 10.1016/j.auec.2021.03.008 34120888 PMC7998048

[38] Birihane BM Bayih WA Tesfahun Y Munye T Alemu AY Belay DM. Health care provider's risk perception, and preparedness towards COVID-19 pandemic in North Central Ethiopia, 2020. Heliyon. (2021) 7:e06610. 10.1016/j.heliyon.2021.e06610 33869847 PMC8035520

[39] Lu W Wang H Lin Y Li L. Psychological status of medical workforce during the COVID-19 pandemic: a cross-sectional study. Psychiatry Res. (2020) 288:112936. 10.1016/j.psychres.2020.112936 32276196 PMC7195354

[40] Ng BH Nuratiqah NA Faisal AH Soo CI Low HJ Najma K. A descriptive study of the psychological experience of health care workers in close contact with a person with COVID-19. Med J Malaysia. (2020) 75:485–9. 32918414

[41] Ge Y Martinez L Sun S Chen Z Zhang F Li F. COVID-19 transmission dynamics among close contacts of index patients with COVID-19: a population-based cohort study in Zhejiang province, China. JAMA Intern Med. (2021) 181:1343–50. 10.1001/jamainternmed.2021.4686 34424260 PMC8383161

[42] Kang L Li Y Hu S Chen M Yang C Yang BX. The mental health of medical workers in Wuhan, China dealing with the 2019 novel coronavirus. Lancet Psychiatry. (2020) 7:e14. 10.1016/S2215-0366(20)30047-X 32035030 PMC7129673

[43] Najwa LZ, Ima, NZ, Wan, MK, Haslinda, NI, Syafinaz, IS, Hasneezah, H. The Concept of District Health Management in Malaysia. Int J Public Health Clin Sci. (2016) 3. Available online at: http://publichealthmy.org/ejournal/ojs2/index.php/ijphcs/article/view/260 (accessed December 30, 2022).

[44] Muhammad Nur Amir AR Binti Amer Nordin A Lim YC Binti Ahmad Shauki NI Binti Ibrahim NH. Workforce mobilization from the national institutes of health for the ministry of health Malaysia: A COVID-19 pandemic response. Front Public Health. (2021) 9:26. 10.3389/fpubh.2021.574135 33643985 PMC7905027

[45] Efstathiou G Papastavrou E Raftopoulos V Merkouris A. Factors influencing nurses' compliance with standard precautions in order to avoid occupational exposure to microorganisms: a focus group study. BMC Nurs. (2011) 10:1. 10.1186/1472-6955-10-1 21255419 PMC3033845

[46] Shawahna R. Knowledge, attitude, and use of protective measures against COVID-19 among nurses: a questionnaire-based multicenter cross-sectional study. BMC Nursing. (2021) 20:163. 10.1186/s12912-021-00689-x 34493274 PMC8422377

[47] Alonzi S La Torre A Silverstein MW. The psychological impact of preexisting mental and physical health conditions during the COVID-19 pandemic. Psychol Trauma. (2020) 12:S236–8. 10.1037/tra0000840 32525380

[48] Fiorillo A Sampogna G Giallonardo V Del Vecchio V Luciano M Albert U. Effects of the lockdown on the mental health of the general population during the COVID-19 pandemic in Italy: results from the COMET collaborative network. Eur Psychiatry. (2020) 63:e87. 10.1192/j.eurpsy.2020.89 32981568 PMC7556907

[49] Somma A Gialdi G Krueger RF Markon KE Frau C Lovallo S. Dysfunctional personality features, non-scientifically supported causal beliefs, and emotional problems during the first month of the COVID-19 pandemic in Italy. Pers Individ Dif. (2020) 165:110139. 10.1016/j.paid.2020.110139 32501318 PMC7247995

[50] World Health Organization. Mental Health and Psychosocial Considerations During the COVID-19 Outbreak, 18 March 2020. World Health Organization (2020). Available online at: https://apps.who.int/iris/handle/10665/331490 (accessed July 6, 2022).

[51] Tam CWC Pang EPF Lam LCW Chiu HFK. Severe acute respiratory syndrome (SARS) in Hong Kong in 2003: stress and psychological impact among frontline healthcare workers. Psychol Med. (2004) 34:1197–204. 10.1017/S0033291704002247 15697046

[52] Beauregard N Marchand A Blanc M-E. What do we know about the non-work determinants of workers' mental health? A systematic review of longitudinal studies. BMC Public Health. (2011) 11:439. 10.1186/1471-2458-11-439 21645393 PMC3141446

