# Supplementary material for: Concerns, perceived impact, practices, preventive measures, and stress among healthcare workers during COVID-19 pandemic in Malaysia
Source: Front Public Health. 2023 Mar 2;11:1028443. doi: 10.3389/fpubh.2023.1028443 (PMC10018122; doi:10.3389/fpubh.2023.1028443)
Supplement: Supplementary file 1 [file Table_1.pdf]

**Table S1.** Concerns, Practice, Preventive Measures, and Impact

| Questions (n=907) | SD (%) | D (%) | A (%) | SA (%) |
|---|---|---|---|---|
| **Concerns (14)** | | | | |
| Close contact with COVID-19 patients | 22 (2.4) | 132 (14.6) | 447 (49.3) | 306 (33.7) |
| The air that I breathe | 40 (4.4) | 243 (26.8) | 449 (49.5) | 175 (19.3) |
| Close contact with colleagues handling COVID-19 patients/samples | 20 (2.2) | 150 (16.5) | 475 (52.4) | 262 (28.9) |
| From objects and surfaces | 21 (2.3) | 150 (16.5) | 496 (54.7) | 240 (26.5) |
| I feel my job puts me a great risk of exposure to COVID-19 | 8 (0.9) | 71 (7.8) | 486 (53.6) | 342 (37.7) |
| I am afraid falling ill with COVID-19 | 22 (2.4) | 144 (15.9) | 460 (50.7) | 281 (31.0) |
| I feel that I should not be looking after patients with COVID-19 | 207 (22.8) | 578 (63.7) | 93 (10.3) | 29 (3.2) |
| I accept the risk of getting COVID-19 as part of my job | 16 (1.8) | 113 (12.5) | 535 (59.0) | 243 (26.8) |
| I find it acceptable if my colleagues resign because of their fear of COVID-19 | 185 (20.4) | 498 (54.9) | 190 (20.9) | 34 (3.7) |
| My family believes that I have a high risk of getting COVID-19 | 22 (2.4) | 120 (13.2) | 537 (59.2) | 228 (25.1) |
| People close to me are at high risk of getting COVID-19 because of my job | 28 (3.1) | 235 (25.9) | 481 (53.0) | 163 (18.0) |
| to my family members | 9 (1.0) | 100 (11.0) | 415 (45.8) | 383 (42.2) |
| to friends | 9 (1.0) | 112 (12.3) | 480 (52.9) | 306 (33.7) |
| People close to me are worried they might get infected with COVID-19 through me | 24 (2.6) | 224 (24.7) | 472 (52.0) | 187 (20.6) |
| **Practice (15)** | | | | |
| Screening of patients and visitors for fever | 2 (0.2) | 11 (1.2) | 286 (31.5) | 608 (67.0) |
| Limiting number of caretakers/visitors | 2 (0.2) | 1 (0.1) | 213 (23.5) | 691 (76.2) |
| Prominent notices to warn patients, caretakers, and visitors | 2 (0.2) | 3 (0.3) | 262 (28.9) | 640 (70.6) |
| Temperature checks for staffs | 3 (0.3) | 16 (1.8) | 312 (34.4) | 576 (63.5) |
| Using 3-ply surgical mask | 1 (0.1) | 15 (1.7) | 272 (30.0) | 619 (68.2 |
| Using N95 face mask | 6 (0.7) | 89 (9.8) | 363 (40.0) | 449 (49.5) |
| Using face shield | 4 (0.4) | 44 (4.9) | 391 (43.1) | 468 (51.6) |
| Using disposable gown | 6 (0.7) | 31 (3.4) | 368 (40.6) | 502 (55.3) |
| Using disposable hair cover | 7 (0.8) | 74 (8.2) | 405 (44.7) | 421 (46.4) |
| Using disposable shoe cover | 7 (0.8) | 86 (9.5) | 413 (45.5) | 401 (44.2) |
| Using alcohol rubs | 1 (0.1) | 6 (0.7) | 280 (30.9) | 620 (68.4) |
| Regular hand washing | 1 (0.1) | 2 (0.2) | 190 (20.9) | 714 (78.7) |
| Special room and area to isolate COVID-19 patients | 1 (0.1) | 2 (0.2) | 162 (17.9) | 742 (81.8) |

| | | | | |
|---|---|---|---|---|
| Changing out of work clothes before going home | 2 (0.2) | 26 (2.9) | 360 (39.7) | 519 (57.2) |
| Showering before going home | 4 (0.4) | 36 (4.0) | 389 (42.9) | 478 (52.7) |
| **Preventive Measures (8)** | | | | |
| I feel that implementation of protective measures at work are generally effective | 2 (0.2) | 20 (2.2) | 413 (45.5) | 472 (52.0) |
| There was adequate training provided to me in the use of PPE | 3 (0.3) | 18 (2.0) | 381 (42.0) | 505 (55.7) |
| I have someone to turn to when I have a problem in using the PPE | 2 (0.2) | 19 (2.1) | 405 (44.7) | 481 (53.0) |
| I feel that the supply of PPE was sufficient | 21 (2.3) | 132 (14.6) | 502 (55.3) | 252 (27.8) |
| Emotional support is available to those who need help (eg. psychological first aid) | 13 (1.4) | 64 (7.1) | 506 (55.8) | 324 (35.7) |
| Clear policies and protocols were instituted for everyone to follow | 5 (0.6) | 25 (2.8) | 485 (53.5) | 392 (43.2) |
| These policies and protocols were implemented quickly enough | 5 (0.6) | 35 (3.9) | 510 (56.2) | 357 (39.4) |
| Most staff have adhered to the recommended measures consistently | 4 (0.4) | 35 (3.9) | 466 (51.4) | 402 (44.3) |
| **Impact (7)** | | | | |
| I have been afraid of telling my family about the risk I am exposed to | 155 (17.1) | 502 (55.3) | 186 (20.5) | 64 (7.1) |
| People avoid me because of my job | 126 (13.9) | 506 (55.8) | 217 (23.9) | 58 (6.4) |
| People avoid my family members because of my job | 194 (21.4) | 563 (62.1) | 118 (13.0) | 32 (3.5) |
| There is more conflict among colleagues at work | 145 (16.0) | 533 (58.8) | 171 (18.9) | 58 (6.4) |
| I feel more stressed at work | 101 (11.1) | 470 (51.8) | 250 (27.6) | 86 (9.5) |
| I have an increase in workload | 42 (4.6) | 235 (25.9) | 446 (49.2) | 184 (20.3) |
| I have to work overtime | 38 (4.2) | 304 (33.5) | 416 (45.9) | 149 (16.4) |

SD = Strongly disagree, D = Disagree, A = Agree, SA = Strongly agree

**Table S2.** IES-R response

| Question | NA (%) | AB (%) | M (%) | QAB (%) | EX (%) |
|---|---|---|---|---|---|
| Any reminder brings back feelings about it | 392 (43.2) | 251 (27.7) | 214 (23.6) | 37 (4.1) | 13 (1.4) |
| Other things kept making me think about it | 451 (49.7) | 224 (24.7) | 200 (22.1) | 23 (2.5) | 9 (1.0) |
| I felt irritable and angry | 510 (56.2) | 234 (25.8) | 133 (14.7) | 20 (2.2) | 10 (1.1) |
| I avoided letting myself get upset when I thought about or was reminded of it | 490 (54.0) | 189 (20.8) | 162 (17.9) | 49 (5.4) | 17 (1.9) |
| I thought about it when I did not mean to | 518 (57.1) | 213 (23.5) | 141 (15.5) | 28 (3.1) | 7 (0.8) |
| Pictures about it popped into my mind | 466 (51.4) | 252 (27.8) | 148 (16.3) | 35 (3.9) | 6 (0.7) |
| I stayed away from reminders of it | 504 (55.6) | 197 (21.7) | 147 (16.2) | 44 (4.9) | 15 (1.7) |
| I was aware that I still had a lot of feeling about it but I did not deal with them | 594 (65.5) | 184 (20.3) | 104 (11.5) | 22 (2.4) | 3 (0.3) |
| I had trouble falling asleep | 627 (69.1) | 156 (17.2) | 89 (9.8) | 24 (2.6) | 11 (1.2) |
| I tried to remove it from my memory | 529 (58.3) | 179 (19.7) | 133 (14.7) | 44 (4.9) | 22 (2.4) |

NA = Not at all, AB = A little bit, M = moderately, QB = Quite a bit, EX= Extremely
